# Highly Sensitive CO-LITES Sensor Based on a Tapered Fiber Focusing and a Fiber-Coupled MPC

**DOI:** 10.3390/s26154828

**Published:** 2026-07-30

**Authors:** Xinhong Yu, Haiyue Sun, Chu Zhang, Runqiu Wang, Shunda Qiao, Ying He, Yufei Ma

**Affiliations:** 1 Zhengzhou Advanced Research Institute, Harbin Institute of Technology, Zhengzhou 450008, China; yuxinhong2026@163.com (X.Y.); zhangchu0717@163.com (C.Z.); 15253997971@163.com (R.W.); shundaqiao@126.com (S.Q.); hearkenyi@163.com (Y.H.); 2 National Key Laboratory of Laser Spatial Information, Harbin Institute of Technology, Harbin 150001, China

**Keywords:** carbon monoxide, tapered fiber, multipass cell, light-induced thermoelastic spectroscopy

## Abstract

In this paper, a highly sensitive carbon monoxide (CO) light-induced thermoelastic spectroscopy (LITES) sensor based on tapered-fiber focusing and a fiber-coupled multipass cell (MPC) is demonstrated for the first time. An MPC with a fiber-coupled structure and an optical length of 40 m is employed to significantly increase the effective absorption path length, reduce the difficulty of optical alignment, and improve the robustness of the sensor system. By comparing five different beam shaping and focusing schemes at the MPC output, the influence of the excitation spot size on the generation of the LITES signal is thoroughly investigated. Experimental results demonstrate that the system response is significantly enhanced as the spot size is compressed. Owing to the micron-scale focused spot produced by the tapered fiber end face, the tapered-fiber configuration exhibits the strongest sensing response among the five schemes. This configuration achieves the highest signal-to-noise ratio (SNR) and exhibits a good linear response to CO concentration. The minimum detection limit (MDL) of the proposed CO-LITES sensor is 3.86 ppm. Based on Allan deviation analysis, when the system integration time reaches 300 s, the minimum detection limit can be further improved to 304 ppb, demonstrating excellent long-term stability.

## 1. Introduction

Carbon monoxide (CO) is an important trace gas in atmospheric chemistry, industrial safety, combustion monitoring, and medical diagnostics. In the atmosphere, CO serves as a major sink for hydroxyl radicals (OH), which are the primary oxidants in the atmosphere. Monitoring CO concentration variations provides valuable insights into atmospheric oxidation capacity and greenhouse gas chemistry [1,2,3,4]. In many industrial processes, the presence of trace amounts of CO may indicate equipment malfunction or potential safety hazards [5,6,7]. Since trace amounts of CO are generated during the smoldering stage of combustion, monitoring CO concentration can facilitate early fire detection. Furthermore, in medical diagnostics, minute variations in exhaled CO concentrations have been associated with asthma, chronic obstructive pulmonary disease, and oxidative stress [8,9,10,11]. Therefore, highly sensitive CO detection is of great importance for environmental monitoring, industrial safety, and medical diagnostics [12,13].

Existing detection technologies, such as electrochemical sensing and semiconductor gas sensing, are relatively mature and widely used, but they still face challenges in stability and selectivity [14,15,16,17,18]. In this context, optical spectroscopic methods have become important tools for trace-gas analysis because of their high sensitivity and molecular specificity. Representative approaches, including mid-infrared QCL spectroscopic systems, self-referenced 2*f*/1*f* WMS schemes, quartz-tuning-fork-assisted laser sensing, and resonant photoacoustic detection, have shown strong potential for CO and other trace-gas measurements [19,20,21,22,23,24,25,26,27,28,29,30,31]. In addition to hardware optimization, recent advances in high-sensitivity gas sensing, particularly those involving intelligent signal processing and self-referenced sensing strategies, have provided useful references for improving trace-gas detection performance. Representative examples include self-referenced spectroscopic methods, QTF-assisted laser sensing, and resonant photoacoustic detection, which have contributed to improvements in sensing sensitivity, selectivity, and measurement stability [32,33]. Among laser absorption spectroscopy-based techniques [34,35,36,37,38,39,40,41,42], quartz-enhanced photoacoustic spectroscopy (QEPAS) has attracted considerable attention owing to its high selectivity and sensitivity [43,44,45,46,47,48]. Nevertheless, QEPAS requires the quartz tuning fork (QTF) to be directly exposed to the target gas, making the QTF vulnerable to corrosion in harsh environments and thereby limiting its practical applications. To address this limitation, light-induced thermoelastic spectroscopy (LITES) has been proposed as a non-contact gas-sensing technique. Unlike QEPAS, LITES eliminates the need to expose the QTF directly to the target gas, thereby improving its applicability in harsh or corrosive environments. In LITES, the laser wavelength is tuned to a characteristic absorption line of the target gas. After passing through the absorption cell, the transmitted laser beam irradiates the surface of the QTF, where the optical energy is absorbed and converted into localized heat. The resulting periodic thermoelastic deformation drives the mechanical resonance of the QTF and generates a piezoelectric signal [49,50,51,52,53,54,55]. Since the amplitude of this signal is related to the gas absorption strength, quantitative detection of the target gas concentration can be achieved [56,57].

The energy coupling condition of the laser beam on the QTF surface is an important aspect of LITES technology, exerting a significant impact on the overall detection performance [58]. To date, most LITES systems have primarily focused on improving the tuning fork structure [59,60]. In addition, in most LITES systems, the excitation beam illuminating the QTF surface is shaped using conventional focusing optics, which limits further reduction in the spot size and thus restricts the achievable localized optical energy density. The thermoelastic signal intensity of the LITES system is closely related to the localized energy density of the beam. A larger laser spot area on the QTF leads to a more dispersed energy distribution of the light field, which results in a gentler thermal gradient inside the QTF and weakens the thermoelastic response amplitude of the system [61,62]. By optimizing the optical focusing system to tightly focus the excitation light into a focused spot, the light field energy can be highly concentrated on the QTF surface. This micro-spot can excite more intense localized thermoelastic deformation, thereby generating a stronger piezoelectric signal response. As the energy transducer in LITES technology, the enhanced response of the QTF can significantly improve the detection sensitivity of the entire sensor system.

In LITES systems pursuing high sensitivity, the multipass cell (MPC) is also a vital component used to increase the interaction length between light and gas. A common MPC consists of two highly reflective concave mirrors. The laser light reflects multiple times between the two mirrors, achieving an equivalent optical path length of tens or even hundreds of meters within a compact physical volume. This significantly extends the effective absorption path, thereby increasing the absorption of the target gas and improving the LITES signal amplitude [63,64]. However, because the MPC relies on the precise reflection of the laser light between the mirror surfaces, small alignment errors can accumulate over multiple reflections, making optical alignment extremely complex. To overcome the deficiencies of traditional systems, such as difficult optical alignment and high susceptibility to environmental disturbances, a fiber-coupled MPC is an effective solution. In this configuration, the beam position and pointing direction are defined by the fiber geometry, which greatly simplifies the alignment process. The laser beam is launched directly from an input fiber into the MPC and subsequently coupled into an output fiber after multiple reflections. This approach effectively improves alignment stability and reduces sensitivity to environmental perturbations.

In this paper, a tapered-fiber focusing strategy is introduced into a fiber-coupled MPC-based CO-LITES sensor to enhance the localized optical excitation of the QTF for the first time. The tapered fiber reduces the beam spot size on the QTF surface, thereby increasing the signal level. Meanwhile, the fiber-coupled MPC extends the absorption path length and reduces the difficulty of optical alignment. Wavelength modulation spectroscopy (WMS) is employed alongside second-harmonic (2*f*) demodulation to extract the target signal, and Allan deviation analysis is performed to evaluate the long-term stability of the system.

## 2. Simulation

**Figure 1:**
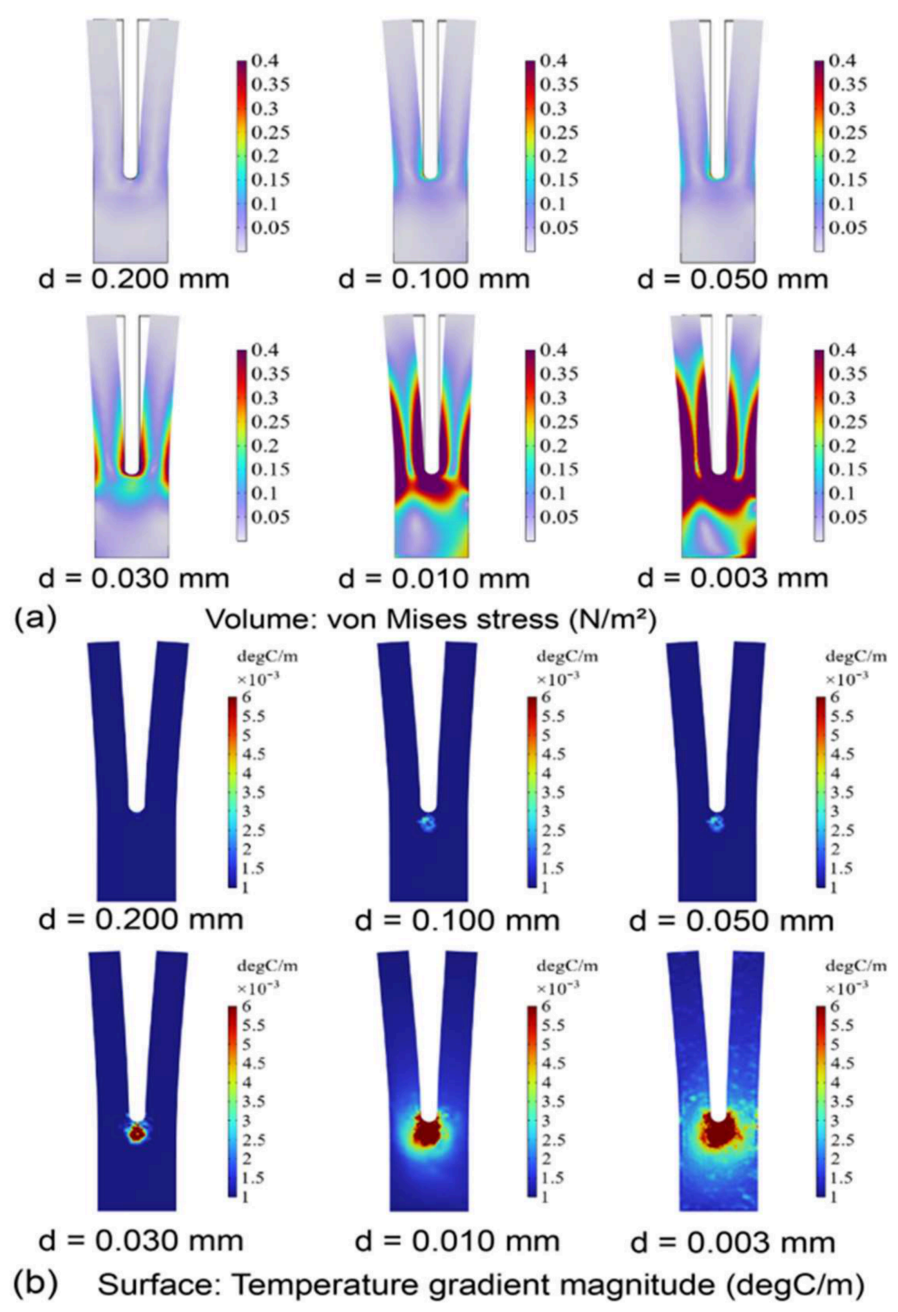
(**a**) Overall stress distribution of the QTF under different laser beam spot diameters; (**b**) Thermal gradient distribution of the QTF under different laser beam spot diameters.

To verify the effect of the spatial distribution characteristics of the laser beam on the surface charge induction process of the QTF, a three-dimensional thermoelastic-piezoelectric coupling model was established using the finite element analysis method. While keeping the total incident laser power constant, the beam spot diameter (d) was gradually reduced from 0.200 mm to 0.003 mm. The overall stress and thermal gradient distributions of the QTF are depicted in Figure 1, respectively. As the beam spot diameter decreases, the local thermal gradient of the QTF is significantly enhanced, leading to a highly concentrated thermoelastic strain. According to the direct piezoelectric effect, the induced electric displacement and corresponding surface charge density are determined by the stress distribution within the QTF. Therefore, a smaller beam spot size excites a more intense piezoelectric response.

**Figure 2:**
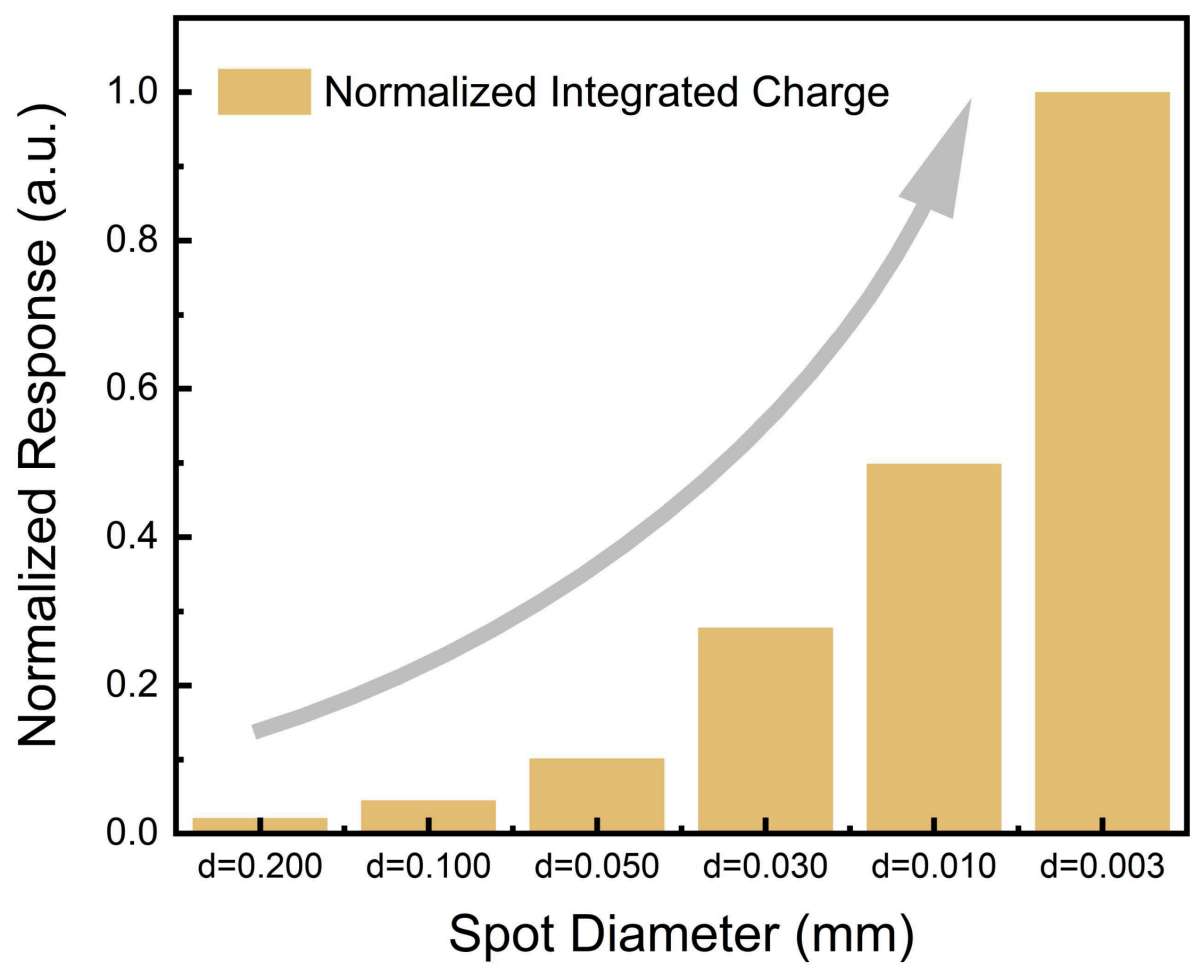
Evolution trend of the normalized total integrated charge as a function of beam spot diameters.

To further quantify this enhancement effect, spatial integration of the surface charge density σ over the effective electrode area was performed to extract the total integrated charge. The dependence of the normalized charge on beam spot diameters is shown in Figure 2. The results indicate that when the laser spot is focused to the micrometer scale, the total piezoelectric charge output of the QTF exhibits significant enhancement.

The analysis reveals that, under a constant incident power, reducing the beam spot size yields a higher energy density at the optimal excitation position of the QTF. This localized stress distribution enhances the effective piezoelectric charge generation, resulting in the integrated charge increasing as the beam spot diameter decreases.

## 3. Experimental Setup

### 3.1. Selecting the CO Absorption Line

**Figure 3:**
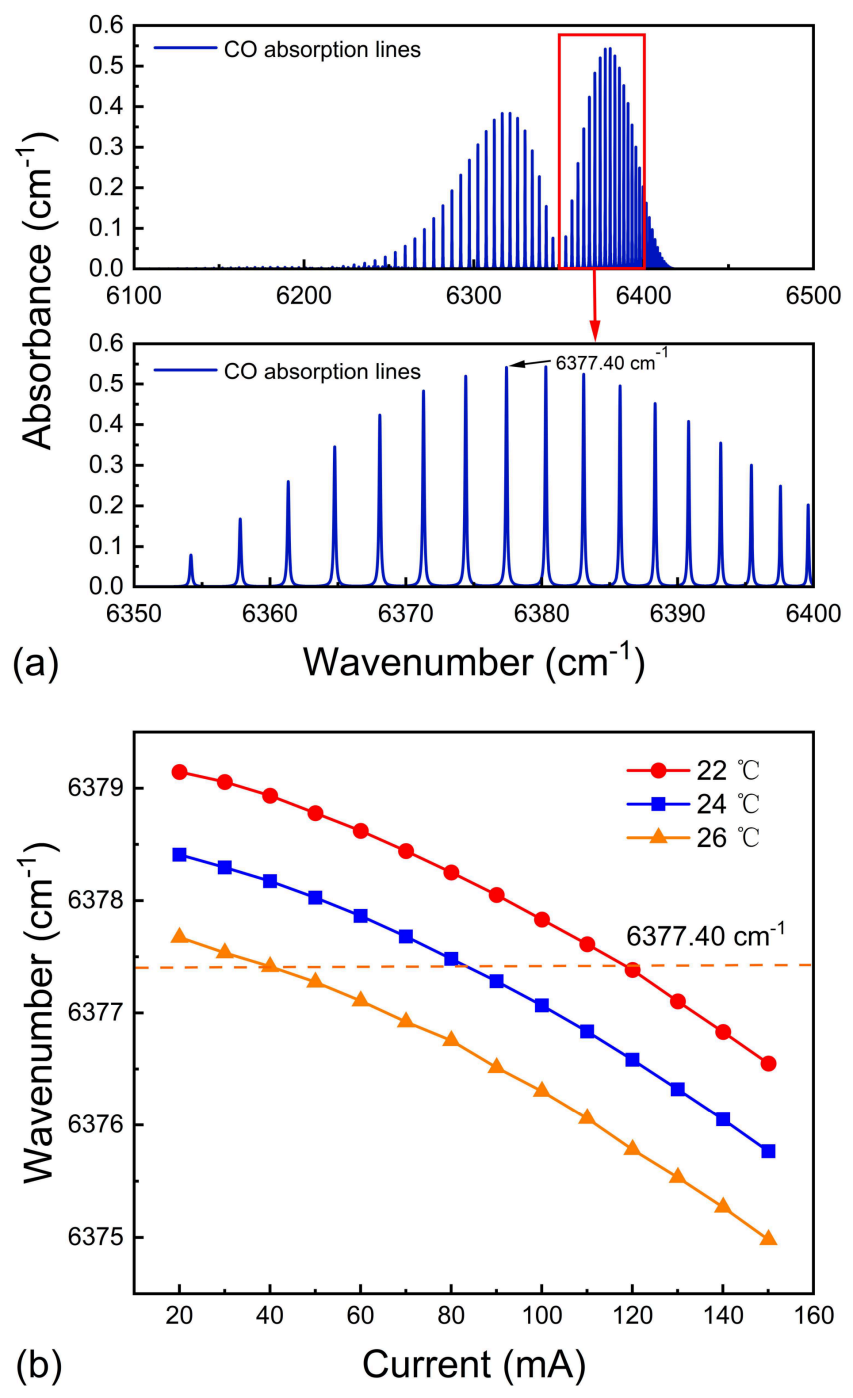
(**a**) CO absorption lines from the HITRAN database; (**b**) Dependence of the laser output wavelength on the injection current at different temperatures.

The selection of the target gas absorption line must satisfy two key criteria: a sufficiently strong absorption line strength and minimal interference from neighboring absorption features of background gases. Based on simulations using the HITRAN database, the line located at 6377.40 cm^−1^ (1568.04 nm) was selected as the target absorption line, as shown in Figure 3a.

A distributed feedback (DFB) diode laser (Model #: SWLD-12782, Wuhan 69 sensor technology Co. Ltd., Wuhan, China) was employed as the light source in this experiment. The variation in its output wavelength with respect to the injection current is depicted in Figure 3b. It is evident that at a constant temperature, the output wavelength increases as the injection current increases. Specifically, at an operating temperature of 24 ℃, the target absorption line can be effectively covered by scanning the injection current centered at approximately 80 mA. 

### 3.2. Experimental Configuration

**Figure 4:**
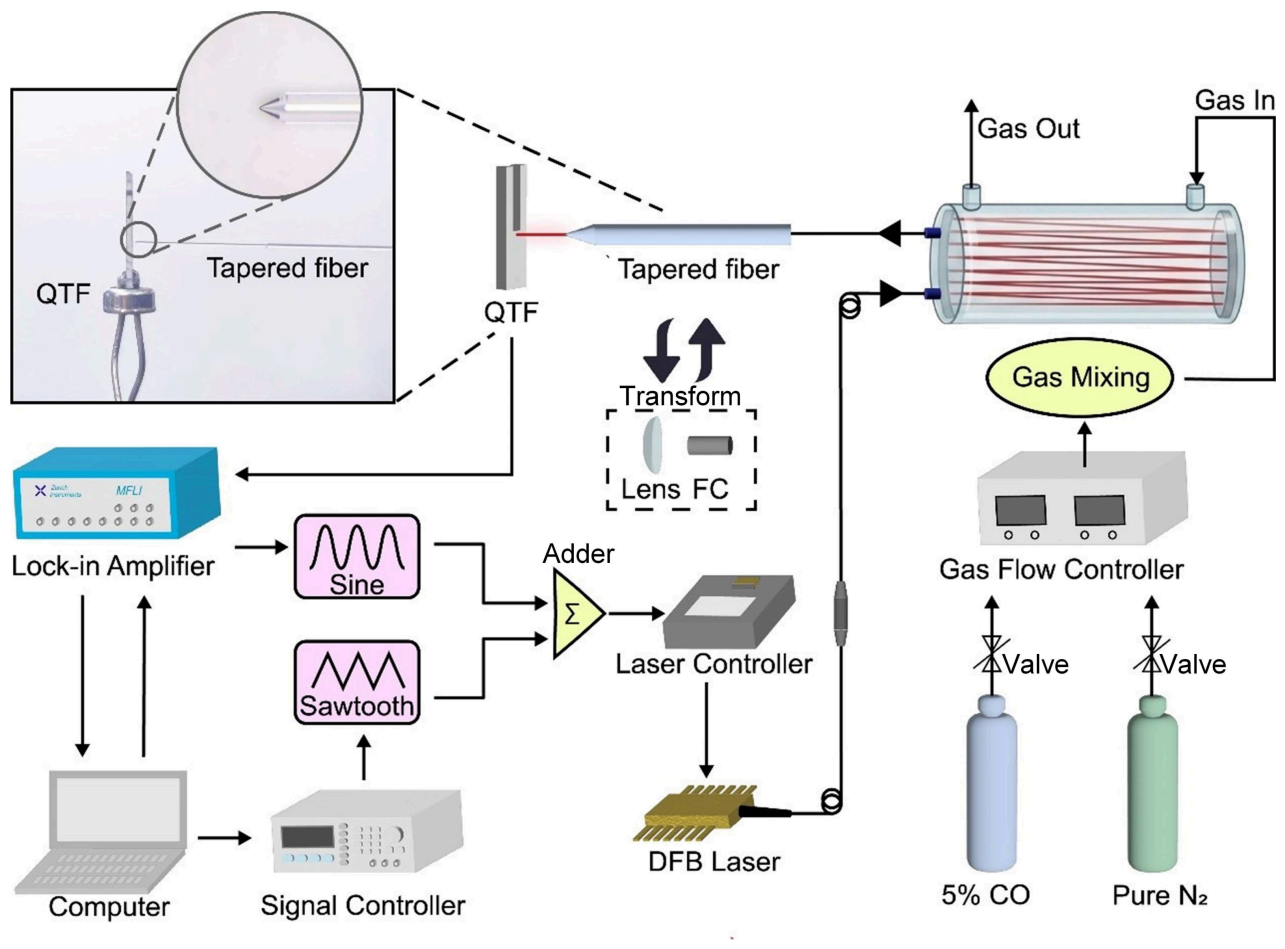
The schematic diagram of the CO-LITES sensor’s experimental setup.

The experimental configuration is illustrated in Figure 4. A DFB diode laser with a central emission wavelength of 1568.04 nm serves as the excitation light source. To extend the effective absorption path length, the beam emitted from the laser output is directly coupled into a fiber-coupled MPC. After exiting the MPC, the transmitted light is shaped and focused using either a fiber collimator combination or a tapered fiber for comparison. Finally, the beam is directed onto the base of the QTF to optimally excite the LITES signal.

To implement signal modulation and data acquisition, the system employs WMS. The laser frequency is simultaneously scanned across the target absorption line by a low-frequency triangular signal from a function generator, and modulated by a high-frequency sinusoidal modulation signal provided by a lock-in amplifier (LIA, Model #: MFLI DC-500 kHz, Zurich Instruments). Following the optical absorption, the weak piezoelectric signal generated by the QTF is routed to the LIA for phase-sensitive demodulation. Finally, the amplitude of the extracted 2*f* signal is utilized to deduce the target gas concentration.

### 3.3. Theoretical Estimation of Excitation Spot Size

To accurately evaluate the effective spot sizes generated by different beam shaping configurations, theoretical calculations were performed. For the lens-based focusing schemes, the output beam from the fiber collimator was regarded as a collimated Gaussian beam. The theoretical beam waist generated by a focusing lens can be expressed as:

(1) $$\omega_{0}=\frac{M^{2}\lambda f}{\pi \omega_{L}}$$

where *ω*_0_ is the beam waist radius at the focal plane, *M*^2^ is the beam quality factor, λ is the laser wavelength, *f* is the focal length of the focusing lens, and *ω_L_* is the incident beam radius on the lens. In this manuscript, the beam waist radius is denoted as *ω*_0_ in the theoretical equations, whereas the corresponding spot diameter *d* = 2*ω*_0_ is used when comparing different focusing configurations.

In accordance with commercial near-infrared fiber collimator specifications, a collimator output beam diameter of 1.5 mm was utilized in the focusing analysis. Therefore, the incident beam radius on the focusing lens was taken as *ω_L_* = 0.75 mm in the theoretical calculation. Under the assumptions of ideal Gaussian beam propagation and *M*^2^ ≈ 1, the theoretically estimated focused spot diameters for the 40 mm, 20 mm, and 10 mm focusing lenses were approximately 53.2 μm, 26.6 μm, and 13.3 μm, respectively. These results indicate that shortening the focal length of the focusing lens can effectively reduce the excitation spot size on the QTF surface and thereby increase the localized optical energy density.

For the tapered fiber, the fiber end face is machined into a conical microlens structure. Due to the refractive index mismatch between the fiber and air, the tapered facet functions as a micro-focusing lens, compressing the emitted beam into a smaller spot area prior to its divergence. Compared with alternative configurations, this conical microlens structure provides superior spatial confinement of the optical field, thereby substantially increasing the localized optical power density on the QTF surface.

The focal-plane waist radius of the tapered fiber can be approximately estimated based on an equivalent microlens model. By considering the curvature radius of the tapered microlens, the focal-plane waist radius can be expressed as:

(2) $$\omega_{0,TF}\approx \frac{M^{2}\lambda R}{\pi \omega_{f}\left(n-1\right)}$$

where *ω*_0,TF_ is the focal-plane waist radius of the tapered fiber, *R* is the curvature radius of the tapered microlens, *ω_f_* is the mode-field radius of the fiber, and *n* is the refractive index of fused silica. In this work, the curvature radius of the tapered microlens was *R* = 6.00 ± 0.05 μm. Using, *ω_f_* ≈ 5.2 μm, the focal-plane waist radius of the tapered fiber was calculated to be approximately 1.3 μm, corresponding to a theoretical focal-plane spot diameter of approximately 2.6 μm.

It should be noted that the spot diameters used in the simulation were defined as Gaussian heat-source diameters, while the spot sizes of the experimental configurations were theoretically estimated based on Gaussian beam propagation or the equivalent microlens model of the tapered fiber. Therefore, the calculated values represent estimated focal-plane spot sizes. Possible uncertainties mainly arise from the beam-quality factor, focal-length tolerance, curvature-radius error of the tapered fiber tip, beam divergence, and the positioning accuracy between the QTF and the focal plane.

## 4. Experimental Results and Discussion

### 4.1. Optimization and Comparison of Laser Beam Shaping Structures

To investigate the influence of the excitation beam spot size on the LITES signal amplitude and to optimize the system structure, this study systematically compares five distinct beam shaping and focusing configurations: fiber collimator only, fiber collimator combined with a 40 mm focusing lens, fiber collimator combined with a 20 mm focusing lens, fiber collimator combined with a 10 mm focusing lens, and a tapered fiber.

For a fair comparison, all five beam-shaping configurations were tested using the same CO concentration, gas pressure, gas flow rate, laser driving parameters, modulation frequency, modulation-depth scanning range, and lock-in amplifier settings. The QTF and MPC were kept unchanged during the comparison, and only the output beam-shaping configuration was replaced.

**Figure 5:**
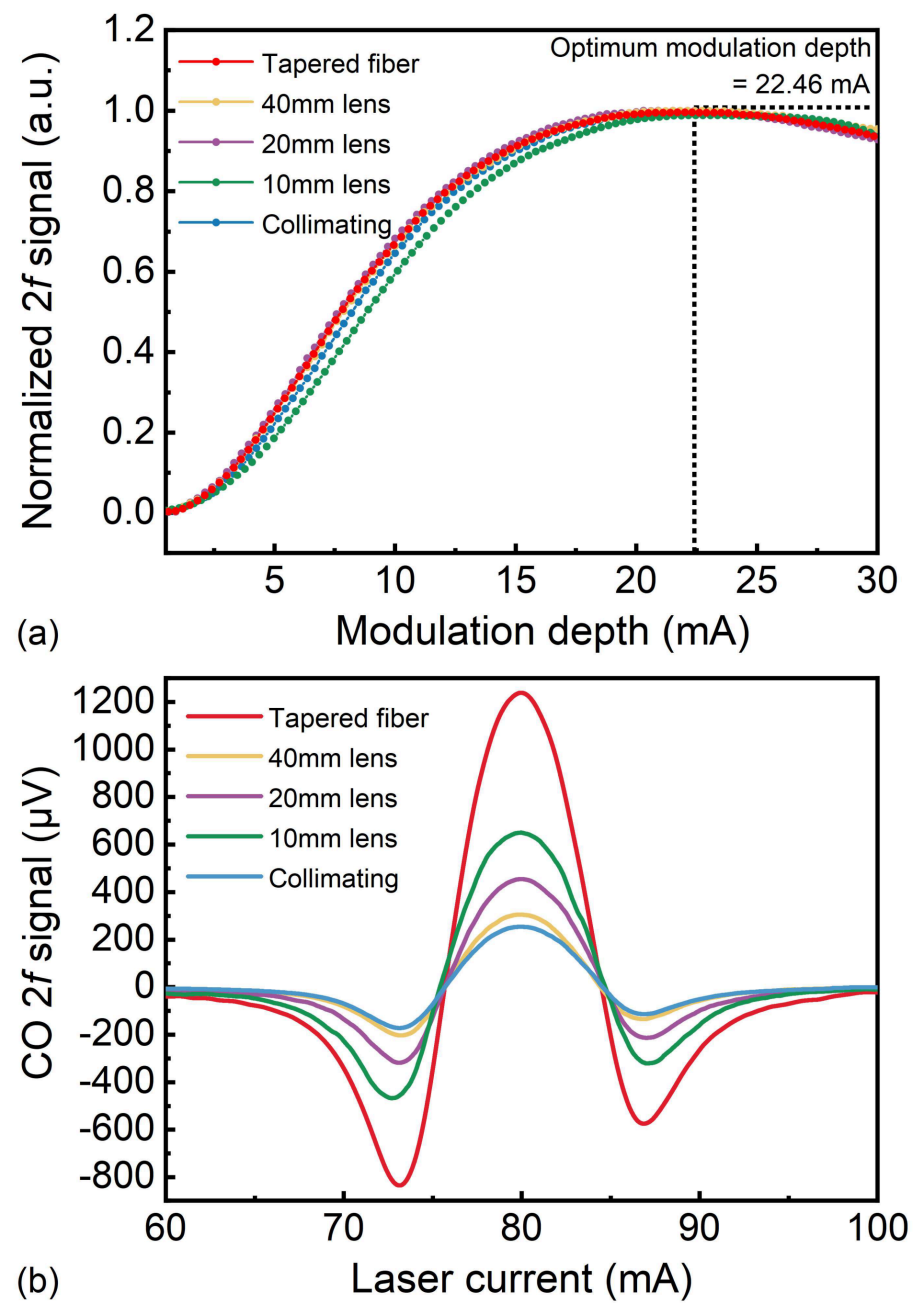
(**a**) 2*f* signal amplitude as a function of modulation depth under five different beam shaping and focusing configurations; (**b**) Measured 2*f* signal profiles at their respective optimal modulation depths. All measurements were performed under the same experimental conditions: temperature of 26 ℃, pressure of 1 atm.

Because the amplitude of the 2*f* signal in WMS strongly depends on modulation depth, optimizing the modulation depth is a prerequisite before conducting performance comparisons. Under identical excitation power and gas concentration, the dependence of the 2*f* signal amplitude on modulation depth for the five optical configurations is shown in Figure 5a. Specifically, as the modulation depth increases, the signal amplitude for each configuration initially increases and subsequently decreases. The optimal modulation depth was experimentally determined to be 22.46 mA for all configurations. Based on this optimized parameter, subsequent comparative experiments clearly demonstrate that the peak amplitudes of the acquired 2*f* signals vary significantly depending on the specific focusing scheme, as depicted in Figure 5b.

**Figure 6:**
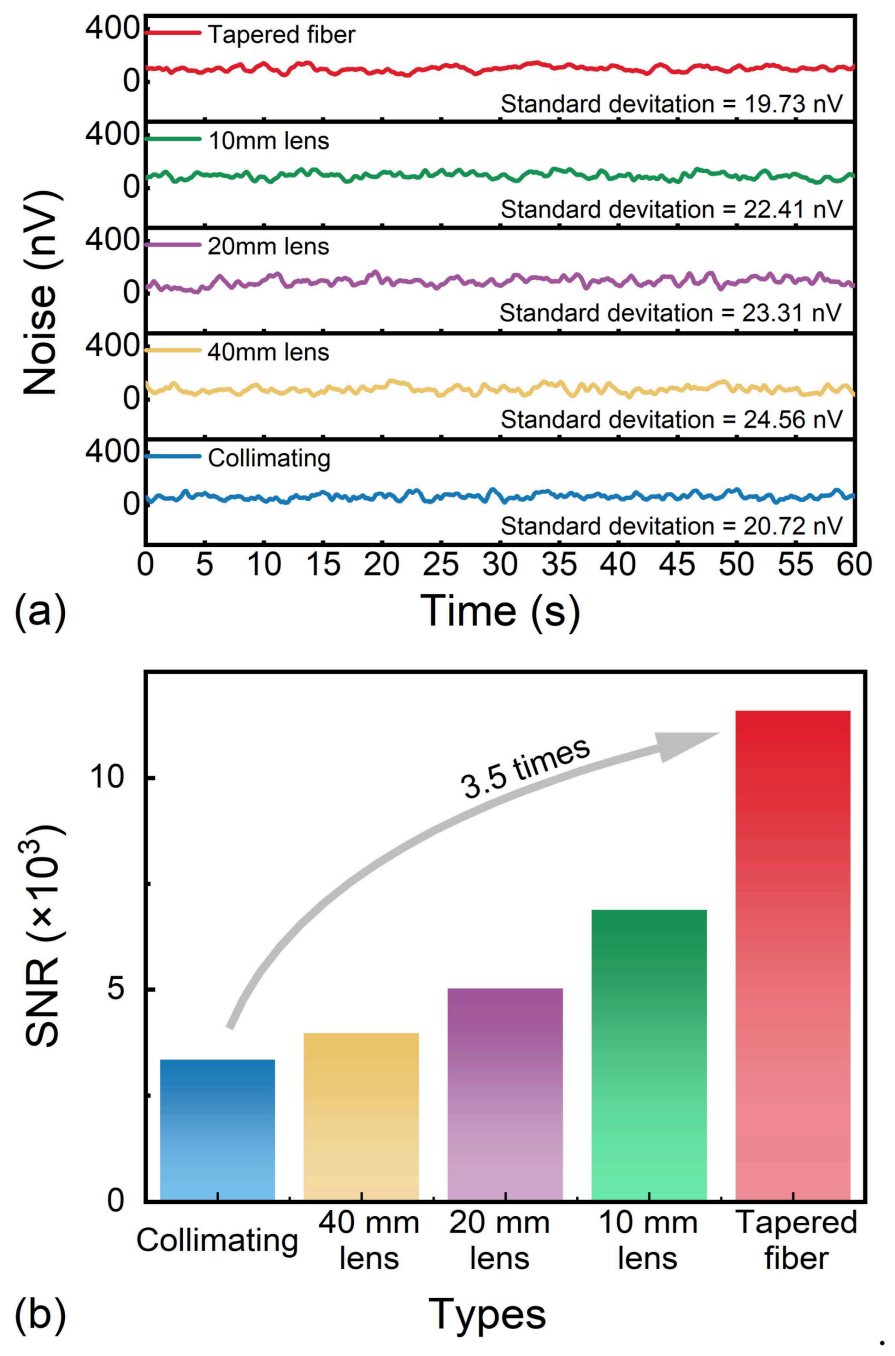
(**a**) Background noise levels under five different beam shaping and focusing configurations; (**b**) SNR comparison under five different beam shaping and focusing configurations.

The background noise levels and the resulting signal-to-noise ratios (SNRs) of the five optical configurations are evaluated, as presented in the bar charts in Figure 6a,b. As the focal length decreases from the bare fiber collimator to the fiber collimator paired with the 10 mm focusing lens, the beam spot is effectively compressed, yielding a significant enhancement in both the 2*f* signal amplitude and the SNR, as clearly observed in Figure 6b. Notably, the configuration employing the tapered fiber achieves the maximum 2*f* signal amplitude of 1.24 mV and the highest SNR. Furthermore, the noise floors across all five configurations remain essentially constant at approximately 100 nV, as depicted in Figure 6a. These results indicate that the observed SNR enhancement primarily originates from the increased signal amplitude enabled by improved optical focusing, rather than from changes in the system noise level.

Both simulation and experimental results indicate that a smaller laser beam spot size significantly enhances the sensor response. However, the experimental enhancement achieved by the tapered fiber is less pronounced than the idealized enhancement predicted by the finite-element simulations. This discrepancy can be attributed to several practical factors. First, the beam emitted from the tapered fiber tip possesses a substantially large divergence angle, causing it to expand rapidly upon exiting the fiber facet. Consequently, even a microscopic working distance between the fiber tip and the QTF surface dictates that the actual beam spot diameter illuminating the QTF is substantially larger than the nominal 2.6 μm. Second, practical factors such as the limited precision of the mechanical translation stage, environmental micro-vibrations, and thermal drift make it highly challenging to continuously and precisely align the micro-scale spot with the optimal charge-accumulation region of the QTF. Even a slight spatial misalignment can lead to a noticeable reduction in signal amplitude. In addition, optical coupling losses may also exist in the practical system. However, in the present fiber-coupled MPC configuration, the input and output optical connections are mainly realized through fiber-to-fiber coupling. Although such connections inevitably introduce minor insertion losses, these losses are relatively small and stable under fixed connection conditions, and therefore have a negligible influence on the comparison among different beam-shaping configurations.

### 4.2. Performance Characterization of the Tapered Fiber LITES Sensor

**Figure 7:**
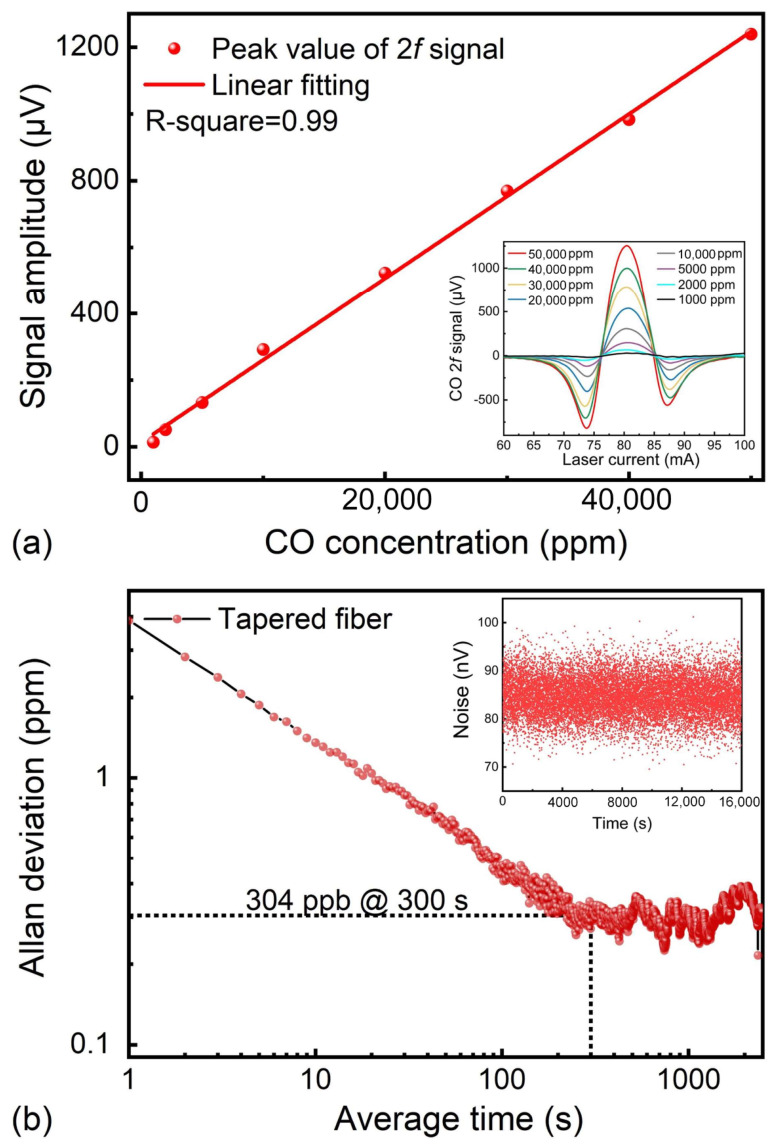
(**a**) The linear relationship between 2*f* signal amplitude and CO concentration. Inset: 2*f* signal of CO-LITES sensor with different concentration; (**b**) Allan deviation analysis of the tapered-fiber-based CO-LITES sensor.

Based on the comparative results above, the configuration utilizing the tapered fiber, which exhibited the optimal performance, was selected for subsequent sensing characterization. First, to evaluate the quantitative analytical capability of the system, its linear response to the target gas at various concentrations was tested. The experimental results demonstrate that the peak amplitude of the 2*f* signal exhibits a highly linear relationship with the gas concentration in the range of 0.1% to 5%, as plotted in Figure 7a. The corresponding linear fitting equation is:

(3) y=0.0247x+13.2352

with an R-squared (R^2^) value of 0.99, where x is the CO concentration in ppm and y is the 2*f* signal amplitude in μV. 

This excellent linearity confirms the suitability of the sensor for quantitative gas analysis. Subsequently, to further assess the long-term stability and the ultimate detection capability of the system, long-term continuous data acquisition was performed in a pure N_2_ environment, and an Allan deviation analysis was conducted, as presented in Figure 7b. The minimum detection limit (MDL) under normal operating conditions was estimated from the measured SNR according to: MDL = C/SNR, where C is the CO concentration used for the SNR measurement and SNR is defined as the ratio between the 2f signal amplitude and the background noise level. Based on the measured SNR at a known CO concentration, the MDL of the tapered-fiber-based CO-LITES sensor was calculated to be 3.86 ppm. According to the Allan deviation analysis, the detection limit can be improved to 304 ppb at an optimal integration time of 300 s. This demonstrates that the system remains highly stable for up to 300 s, effectively suppressing the low-frequency noise induced by environmental perturbations and laser drift, demonstrating its potential for high-performance and stable gas-sensing applications.

## 5. Conclusions

A highly sensitive CO-LITES sensor based on a tapered fiber focusing and a fiber-coupled MPC is proposed for the first time. The influence of the excitation beam spot size on LITES signal generation is studied by systematically comparing five different beam shaping and focusing schemes. The experimental results reveal that the system response is significantly enhanced as the beam spot size is compressed. Benefiting from its micron-scale tight-focusing end face, the tapered fiber achieves the best sensing response among the five schemes. This configuration yields the highest 2*f* signal amplitude and the highest SNR without introducing additional system background noise. In the system performance characterization, the tapered-fiber-based LITES sensor exhibits excellent linearity within the concentration range of 0.1–5%, with a linear fitting coefficient R^2^ of 0.99, confirming its suitability for quantitative gas analysis. Furthermore, under normal operating conditions, the MDL of the system was determined to be 3.86 ppm. Based on Allan deviation analysis, the detection limit could be improved to 304 ppb at an integration time of 300 s, demonstrating the excellent long-term stability and drift-suppression capability of the sensor system. Overall, this work provides a meaningful route for improving LITES performance through optical energy coupling rather than only modifying the QTF structure. The combination of tapered-fiber focusing and a fiber-coupled MPC enhances localized excitation while maintaining alignment robustness, which is beneficial for practical trace-gas sensing applications.

## Data Availability

The data presented in this study are available on request from the corresponding author.
